# Microwave-Assisted Synthesis and Biological Evaluation of Dihydropyrimidinone Derivatives as Anti-Inflammatory, Antibacterial, and Antifungal Agents

**DOI:** 10.1155/2013/197612

**Published:** 2013-04-15

**Authors:** Anjna Bhatewara, Srinivasa Rao Jetti, Tanuja Kadre, Pradeep Paliwal, Shubha Jain

**Affiliations:** Laboratory of Heterocycles, School of Studies in Chemistry and Biochemistry, Vikram University, Ujjain, Madhya Pradesh 456010, India

## Abstract

A simple protocol for the efficient preparation of aryl and heteroaryl substituted dihydropyrimidinone has been achieved via initial Knoevenagel, subsequent addition, and final cyclization of aldehyde, ethylcyanoacetate, and guanidine nitrate in the presence of piperidine as a catalyst in solvent-free under microwave irradiation. The synthesized compounds showed a good anti-inflammatory, antibacterial, and antifungal activity.

## 1. Introduction

Pyrimidinones have been paid increasing attention, due to their various therapeutic and pharmacological properties, such as antiviral, antibacterial, antihypertensive, and antitumor effects [1]. More recently, they emerged as integral backbones of several calcium blockers, antihypertensive agents, *α*-1a-antagonists, and neuropeptide Y (NPY) antagonists [2]. Pyrimidinone derivatives are found as core units in many marine alkaloids (batzelladine and carambine), which have been found to be potent to HIV-gp-120 CD4 inhibitors [3].

Due to the remarkable biological utilization, the pyrimidinones attract many researchers as well as academicians. Recently, several methods improved the procedure using phosphorus pentoxide-methanesulfonic acid [4], potassium *ter*-butoxide (*t*-BuOK) [5], ammonium dihydrogen phosphate [6], silica-gel [7], mesoporous molecular sieve MCM-41 [8], cyanuric chloride [9], nano-BF_3_ *·*SiO_2_ [10], silica gel-supported polyphosphoric Acid [11], zirconium(IV) chloride [12], and indium(III) bromide [13] as catalysts. However, some of these one-pot procedures generally require strong protic or Lewis acids, prolonged reaction times, and high temperature. Consequently, there is a scope for further modification towards mild reaction condition, increased variation of the substituents, and improved yields.

Microwave promoted solvent-free reactions [14] are well known as environmentally benign methods that also usually provide improved selectivity, enhanced reaction rates, cleaner products, and manipulative simplicity [15]. However, these procedures are practically limited as the solvents in microwave oven at elevated temperatures create high pressures, which may cause explosion. To circumvent these problems, there is a need for the development of newer methods which proceed under mild and solvent free condition. 

Nowadays solvent-free reactions gained much importance in organic synthesis because of the high yields and shorter reaction times. Earlier reported procedures for the synthesis of pyrimidine derivatives typically involved longer reaction time and fewer yields [16]. In the present communication, we would like to describe the advantages of dry reaction techniques coupled with microwave activation and their applications to organic synthesis [17]. 

## 2. Experimental

### 2.1. General

All reactions were carried out in an LG domestic unmodified microwave oven model MS-1947C/01. Reactions were routinely monitored by thin layer chromatography (TLC) on silica gel (precoated F 254 Merck plates) and visualized the products under UV light (254 nm). ^1^H NMR spectra were determined in CDCl_3_ or DMSO-*d*_6_ solutions with a Bruker Advance II 400 MHz spectrometer and signals recorded in parts per million (*δ*) downfield from tetramethylsilane as internal standard. IR spectra were recorded on Perkin Elmer FT-IR spectrometer (Spectrum RX I) using KBr pellet technique. The elemental analyses were performed using thermo EA 2110 series. Melting points were recorded in open capillaries on LABINDIA melting point apparatus and were uncorrected. Mass spectra (ESI) were recorded on Waters Micromass Q-TOF Micro. Anti-inflammatory activity has been carried out in Institute of Pharmacy, Vikram University, Ujjain. 

### 2.2. General Procedure for the Synthesis of 2-amino Dihydropyrimidinone Derivatives **4(a-h)**

A mixture of aldehyde **1** (1 mmol), ethyl cyanoacetate **2** (1.2 mmol), guanidine nitrate **3** (1.5 mmol), and 2-3 drops of piperidine was subjected to microwave irradiation at 60% power in 600 W microwave oven for 5 min. (Successive irradiation of 30–40 sec with cooling intervals of time, the temperature being 90–100°C). On completion of reaction, indicated by TLC, the mixture was cooled and quenched with water (3 × 10 mL). The solid product was separated and recrystallized from ethanol to afford pure products **4(a-h)** in good yields.

### 2.3. Spectral Data

*2-amino-4-oxo-6-phenyl-1,4,5,6-tetrahydropyrimidine-5-carbonitrile* **(4a)**. White crystals; Mp: 218–220°C; IR (KBr *v*_max_/cm^−1^): 3478 (NH), 3090 (C–H), 2260 (CN), 1690 (C=O), 1617 (C=N), 1567 (C=C). ^1^H NMR (300 MHz, CDCl_3_) *δ* 8.56, 2.0 (s, 2H NH), 7.27–7.40 (m, 5H, Ar), 4.1 (d, CH, *J* = 8.4 Hz), 3.97 (d, CH, *J* = 11.5 Hz). ^13^C NMR (CDCl_3_) *δ*: 168.4, 153.3, 143.5, 128.5, 126.9, 126.7, 116.8, 43.2, 42.4. Molecular weight: 214.22; Mass (*m*/*z*): 214 (M^+^); C_11_H_10_N_4_O (214.09); Cacld. C, 61.67; H, 4.71; N, 26.15; O, 7.47; Found. C, 60.07; H, 4.13; N, 25.87; O, 7.08.

*2-amino-6-(4-methoxyphenyl)-4-oxo-1,4,5,6-tetrahydropyrimidine-5-carbonitrile* **(4b)**. Light yellow crystals; Mp: 110–112°C; IR (KBr *v*_max_/cm^−1^): 3318 (NH), 3055 (C–H), 2190 (CN), 1710 (C=O), 1620 (C=N), 1565 (C=C). ^1^H NMR (300 MHz, CDCl_3_) *δ*: 8.32 (s, 2H, NH_2_), 6.94–7.18 (m, 4H, Ar), 3.9 (d, CH, *J* = 14.4 Hz), 3.72 (d, CH, *J* = 11.5 Hz), 3.50 (s, 3H, OCH_3_), 2.0 (s, 2H NH). ^13^C NMR (CDCl_3_) *δ*: 166.7, 159.1, 153.5, 136.2, 127.4, 116.3, 115.0, 56.1, 43.6, 44.8. Molecular weight: 244; Mass (*m*/*z*): 243 (M^+^); C_12_H_12_N_4_O_2_ (244.10); Cacld. C, 59.01; H, 4.95; N, 22.94; O, 13.10; Found. C, 58.87; H, 4.63; N, 22.65, O, 12.72.

*2-amino-6-(3,4-dimethoxyphenyl)-4-oxo-1,4,5,6-tetrahydropyrimidine-5-carbonitrile* **(4c)**. Yellow crystals; Mp: 127-128°C; IR (KBr *v*_max_/cm^−1^): 3460 (NH), 3080 (arom. C–H), 2943 (methyl C–H), 1590 (C=C), 1290 (aryl OCH_3_), 1670 (C=O), 1567 (C=N), 2310 (CN).^ 1^H NMR (300 MHz, CDCl_3_) *δ*: 6.74–6.96 (m, 3H, Ar), 2.0, 8.56 (s, 2H, NH), 3.97 (d, CH, *J* = 8.4 Hz), 4.1 (d, CH, *J* = 11.5 Hz), 3.83 (s, 2H, methylene proton). ^13^C NMR (CDCl_3_) *δ*: 168.4, 153.3, 149.6, 147.8, 136.8, 121.9, 118.9, 116.8, 109.8, 43.2, 42.7, 56.1. Molecular weight: 274.28; Mass (*m*/*z*): 248 (M^+^); C_13_H_14_N_4_O_3_ (274.11); Cacld. C, 56.93; H, 5.14; N, 20.43; O, 17.50; Found. C, 56.14; H, 5.04; N, 21.98; O, 17.19.

*2-amino-6-(4-nitrophenyl)-4-oxo-1,4,5,6-tetrahydropyrimidine-5-carbonitrile* **(4d)**. Yellow crystals; Mp: 162–164°C; IR (KBr *v*_max_/cm^−1^): 3440 (NH), 3080 (C–H), 2327 (CN), 1640 (C=O), 1560 (C=N), 1567 (C=C), 1523 (N=O). ^1^H NMR (300 MHz, CDCl_3_) *δ*: 7.55–8.21 (dd, 4H, Ar), 2.0, 8.56 (s, 2H, NH), 3.97 (d, CH, *J* = 11.5 Hz), 4.1 (d, CH, *J* = 8.4 Hz). ^13^C NMR (CDCl_3_) *δ*: 168.4, 153.3, 149.6, 145.9, 123.7, 123.4, 116.8, 43.2, 42.4. Molecular weight: 259.22; Mass (*m*/*z*): 259 (M^+^); C_11_H_9_N_5_O_3_ (259.07); Cacld. C, 50.97; H, 3.50; N, 27.02; O, 18.52; Found. C, 50.73; H, 3.24; N, 26.92; O, 18.32. 

*2-amino-4-oxo-6-(1H-pyrrol-2-yl)-1,4,5,6-tetrahydropyrimidine-5-carbonitrile* **(4e)**. Yellow crystals; Mp: 93–95°C; IR (KBr *v*_max_/cm^−1^): 3420 (NH), 3111 (Ar C–H), 2360 (CN), 1720 (C=O), 1593 (C=N). ^1^H NMR (300 MHz, CDCl_3_) *δ*: 5.72–6.69 (m, 3H, Ar), 2.0, 8.56 (s, 2H, NH), 3.02 (m, 1H, CH), 3.9 (m, 1H, CH). ^13^C NMR (CDCl_3_) *δ*: 168.4, 153.3, 130.5, 118.0, 116.8, 108.5, 107.7, 43.9, 42.8. Molecular weight: 203.20; Mass (*m*/*z*): 203 (M^+^); C_9_H_9_N_5_O (203.08); Cacld. C, 53.20; H, 4.46; N, 34.47; O, 7.87; Found. C, 52.84; H, 4.13; N, 33.94; O, 7.59. 

*2*-*amino-6-(furan-2-yl)-4-oxo-1,4,5,6-tetrahydropyrimidine-5-carbonitrile* **(4f)**. Light yellow crystals; Mp: 76–78°C IR (KBr *v*_max_/cm^−1^): 3427 (NH), 3121 (C–H), 2230 (CN), 1690 (C=O), 1615 (C=N), 1572 (C=C). ^1^H NMR (300 MHz, CDCl_3_) *δ*: 7.92 (s, 2H, NH_2_), 6.54–7.67 (m, 3H, Ar), 4.1 (d, CH, *J* = 11.5 Hz), 3.82 (d, CH, *J* = 8.4 Hz), 2.0 (s, 2H, NH). ^13^C NMR (CDCl_3_) *δ*: 164.5, 153.6, 149.9, 142.1, 116.5, 111.1, 110.4, 44.3, 40.7. Molecular weight: 204; Mass (*m*/*z*): 203 (M^+^); C_9_H_8_N_4_O_2_ (204.06); Cacld. C, 52.94; H, 3.95; N, 27.44; O, 15.67; Found. C, 52.81; H, 3.78; N, 26.97; O, 15.41. 

*2*-*amino-6-(1H-indol-3-yl)-4-oxo-1,4,5,6-tetrahydropyrimidine-5-carbonitrile* **(4g)**. Yellow crystals; Mp: 190–192°C; IR (KBr *v*_max_/cm^−1^): 3450 (NH), 3120 (C–H), 2310 (CN), 1690 (C=O), 1590 (C=N), 1534 (C=C). ^1^H NMR (300 MHz, CDCl_3_) *δ*: 10.8 (s, 1H, NH), 8.22 (s, 2H, NH_2_), 7.11–7.69 (m, 5H, Ar), 3.7 (d, CH, *J* = 14.0 Hz), 3.4 (d, CH, *J* = 8.0 Hz), 2.0 (s, 2H, NH). ^13^C NMR (CDCl_3_) *δ*: 164.3, 154.1, 133.2, 126.4, 124.3, 122.5, 120.6, 114.7, 117.1, 110.9, 43.1, 45.4. Molecular weight: 253; Mass (*m*/*z*): 252 (M^+^); C_13_H_11_N_5_O (253.10); Cacld. C, 61.65; H, 4.38; N, 27.65; O, 6.32; Found. C, 61.16; H, 3.99; N, 27.34; O, 5.89.

*2-amino-4-oxo-6-(1-methyl-1H-pyrrol-2-yl)-1,4,5,6-tetrahydropyrimidine-5-carbonitrile* **(4h)**. Yellow crystals; Mp: 148–150°C; IR (KBr *v*_max_/cm^−1^): 3470 (NH), 3111 (arom. C–H), 2360 (CN), 1720 (C=O), 1542 (C=C), 1593 (C=N), 2953 (methyl C–H). ^1^H NMR (300 MHz, CDCl_3_) *δ*: 5.72–6.69 (m, 3H, Ar), 2.0, 8.56 (s, 2H, NH), 3.90 (s, 1H, CH_3_), 3.97 (d, CH, *J* = 8.4 Hz), 4.1 (d, CH, *J* = 11.5 Hz). ^13^C NMR (CDCl_3_) *δ*: 168.4, 153.3, 132.1, 122.5, 116.8, 108.6, 108.4, 44.2, 40.3, 35.2. Molecular weight: 217.23; Mass (*m*/*z*): 217 (M^+^); C_10_H_11_N_5_O (217.10); Cacld. C, 55.29; H, 5.10; N, 32.24; O, 7.37; Found. C, 54.94; H, 4.92; N, 31.91; O, 7.03.

### 2.4. Pharmacology

Colony bred healthy rats of Wistar strain and albino mice procured from local market from Ujjain were used for the study. They were housed in standard polypropylene cages under room temperature (24 ± 2°C), relative humidity (60%–70%), and exposed to 12 : 12 hours light : dark cycle. The rats were fed Nutrilab Rodent Feed and drinking water filtered through an Aqua guard water filter system *adlibitum. *They were allowed to acclimatize for 5 days prior to commencement of dosing. The protocol was ethically approved by IAEC of the institute.

### 2.5. Anti-Inflammatory Activity

The anti-inflammatory activity was determined in vivo [18] using the carrageenan-induced rat paw edema test [5, 11]. A solution of 0.1 mL of 1% carrageenan (Sigma-Aldrich, Dorset, UK) in saline was injected subplantarly in the right hind paw of the rats 1 h after IP administration of compounds. The paw thickness was measured from the ventral to the dorsal surfaces using a dial caliper immediately prior to carrageenan injection and then at each hour, up to 4 h after the subplanar injection. The edema was calculated as the thickness variation between the carrageenan and saline treated paw. Anti-inflammatory activity was expressed as the percent of inhibition of the edema when compared with the control group. The results are expressed as the mean ± SEM of *n* animals per group. The data was statistically analyzed by one way analysis of variance (ANOVA) followed by Tukey multicomparison test. Differences with *P* < 0.05 between experimental groups were considered statistically significant.

### 2.6. Antibacterial Activity

Antibacterial activity of the prepared compounds **4d, 4e, 4f, 4g**, and **4h** were tested by the disk diffusion method [19]. Whattman no. 1 filter paper disks were sterilized by autoclaving for 1 h at 140°C. The sterile disks were impregnated with the test compounds (250 mg/mL). Agar plates were uniformly surface inoculated with fresh broth culture of *Staphylococcus aureus, Basillus subtilis, Escherichia coli*, and* Pseudomonas aeruginosa.* The impregnated disks were placed on the medium suitably spaced apart, and the plates were incubated at 30°C for 1 h to permit good diffusion and were then transferred to an incubator at 37 ± 2°C for 24 h. The zones of inhibition were measured on mm scale. The results of antimicrobial activity tests are listed in Table 3.

### 2.7. Antifungal Activity

Antifungal susceptibility test was done by disk diffusion method [20] using Sabouraud's dextrose agar medium. After sterilization, the medium was inoculated with *Candida albicans, Aspergillus flavus*, and *Aspergillus niger.* The standard antifungal agent clotrimazole (100 g/mL), solvent control (0.5% v/v Tween 80), and the newly synthesized compounds **4d**, **4e**, **4f**, **4g**, and **4h** in a concentration of 100 *μ*g/mL were then added by sterile micropipette. The plates were then incubated at 37°C for 24 h and the diameter of zone of inhibition was measured and recorded in Table 3.

## 3. Results and Discussion

**Scheme 1:**
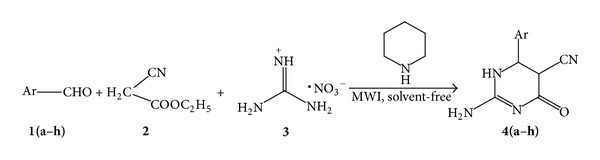


In the view of the above mentioned limitations of the reported method, pharmacological importance of dihydropyrimidinones and our ongoing endeavors to conduct organic synthesis under solvent free conditions [21], we describe an expeditious solvent less microwave accelerated approach for the rapid assembly of 2-amino dihydropyrimidinones. Aromatic aldehydes (**1a**–**h**, 1 mmol) on reaction with ethyl cyanoacetate (**2**, 1.2 mmol) and guanidine nitrate (**3**, 1.5 mmol) using dry conditions yielded corresponding 2-amino dihydropyrimidinones (Scheme 1).

As far as our interest in investigating the facile, rapid, and expeditious solvent-less methodology for 2-amino dihydropyrimidinone, we tried the reaction of benzaldehyde (**1a**, 1 mmol), with ethyl cyanoacetate (**2**, 1.2 mmol) and guanidine nitrate (**3**, 1.5 mmol) by varying microwave power from 150 watts to 750 watts. It was observed that by increase in power up to 600 watts, there was increase in yield and shortened reaction time. Beyond the 600 watts there was no significant change in reaction time and yield. 

In order to evaluate the generality of this model reaction, we then prepared a range of 2-amino dihydropyrimidinone derivatives under the optimized reaction conditions. In all cases, aryl aldehydes and heteroaryl aldehydes with substituents carrying either electron-donating or electron-withdrawing groups reacted successfully and gave the expected products in good to excellent yields in relatively short reaction times. The kind of aldehyde has no significant effect on the reaction. The results are shown in Table 1.

In the presence of piperidine, reaction proceeds smoothly giving desired products in short time and in a good yield. The formation of the product takes place when aryl aldehydes were reacted with ethyl cyanoacetate to form arylmethylene ethylcyanoacetate, which subsequently added with guanidine followed by cyclization and tautomerization to form the desired product.

 The results of the anti-inflammatory activity of compounds **4d, 4e, 4f, 4g, and 4h** were summarized in Table 2. From the results, it is evident that compounds **4c**, **4f**, and **4h** as well as indomethacin as the reference drug induced significant anti-inflammatory activity after 3 and 4 h in comparison to control and almost all of the tested compounds were shown moderate to good anti-inflammatory activity. 

The MIC values of the test solutions are recorded in Table 3 which is recorded in zones of inhibition in mm for the bacteria and fungi.

The antimicrobial screening has shown that compounds **4d**, **4f, 4g**, and** 4f **have displayed moderate activity against gram +ve bacteria tested, that is, *S. aureus *and *B. subtilis. *Compounds **4d** and **4h** have shown broad spectrum activity against gram −ve bacteria tested, that is, *E. coli *and* P. aeruginosa *while compound **4e** has shown poor activity against both gram +ve and gram −ve bacteria tested.

Compound **4d **has shown good antifungal activity against all the tested fungi, that is, *C. albicans, A. flavus*, and* A. niger* while the remaining compounds **4e** and **4f** have shown good activity, and compounds **4g** and **4h** have shown moderate activity against all fungi tested.

## 4. Conclusion

 In summary, we have described one-pot synthesis of 2-amino dihydropyrimidinone derivatives via a three component cycloaddition reaction under microwave irradiation. Another advantage of this method is excellent yields in shorter reaction time with high purity of the products. The synthesized compounds have shown good anti-inflammatory, antibacterial, and antifungal activities.

## Figures and Tables

**Table 1:** Piperidine catalyzed synthesis of 2-amino dihydropyrimidinones.

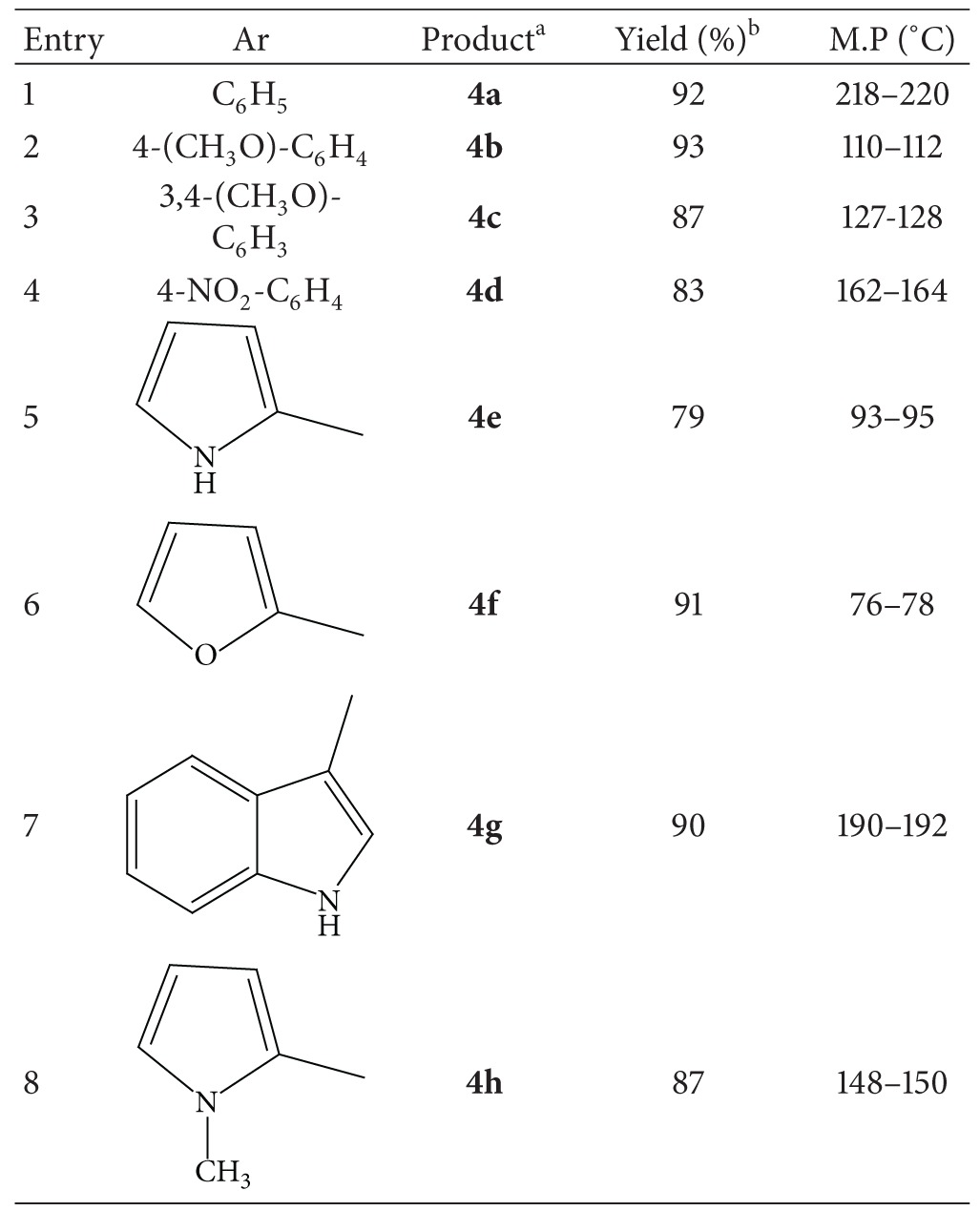

^a^All compounds thus obtained were characterized by physical and spectral data. ^b^Isolated yields.

**Table 2:** Effects of compounds (**4d**, **4e**, **4f**, **4g**, and **4h**) and indomethacin in the inhibition of carrageenan-induced rat paw edema.

Entry	Drug	Dose	Paw volume response at different time intervals in mean ± SEM			
			1 hour	2 hour	3 hour	4 hour
1	Control	10 mL/kg	0.5912 ± 0.005∗∗∗	0.5930 ± 0.002∗∗∗	0.6102 ± 0.002∗∗∗	0.6303 ± 0.002∗∗∗
2	Indomethacin (standard)	100 mg/kg	0.2700 ± 0.005∗∗ (54.33%)	0.3124 ± 0.005∗∗ (47.31%)	0.3133 ± 0.008∗∗ (48.65%)	0.3500 ± 0.005∗∗ (44.47%)
3	**4d**	20 mg/kg	0.4000 ± 0.005∗∗∗ (32.34%)	0.4300 ± 0.005∗∗ (27.48%)	0.4500 ± 0.011∗∗∗ (26.25%)	0.4711 ± 0.011∗∗∗ (25.25%)
4	**4e**	40 mg/kg	0.3533 ± 0.003∗∗ (40.24%)	0.3833 ± 0.003∗∗ (35.36%)	0.3967 ± 0.003∗∗ (34.98%)	0.4200 ± 0.010∗∗ (33.36%)
5	**4f**	20 mg/kg	0.4133 ± 0.003∗∗ (30.09%)	0.4433 ± 0.003∗∗ (25.24%)	0.4667 ± 0.008∗∗∗ (23.51%)	0.4933 ± 0.012∗∗∗ (21.73%)
6	**4g**	40 mg/kg	0.3567 ± 0.003∗∗ (39.66%)	0.3867 ± 0.003∗∗ (34.78%)	0.4133 ± 0.003∗∗ (32.26%)	0.4520 ± 0.005∗∗∗ (28.28%)
7	**4h**	20 mg/kg	0.3833 ± 0.003∗∗ (35.16%)	0.4133 ± 0.003∗∗∗ (30.30%)	0.4433 ± 0.003∗∗ (27.35%)	0.4667 ± 0.008∗∗∗ (25.95%)

Values are expressed as mean ± SEM; *n* = 6 in each group, ∗∗∗*P* < 0.001, ∗∗*P* < 0.01, compared to control. Data was analysed by one way ANOVA followed by Duneet's test. Formula % inhibition = *V*_*c*_ − *V*_*t*_/*V*_*c*_ × 100 (*V*-volume, *C*-control, *t*-test).

**Table 3:** Antimicrobial evaluation of synthesized compounds.

Compound	Norfloxacin	**4d**	**4e**	**4f**	**4g**	**4h**
Gram +ve bacteria						
*S. aureus ATCC 25922 *	20	18	10	16	15	18
*B. subtilis ATCC 6633 *	22	17	12	15	16	17
Gram −ve bacteria						
*E. coli ATCC 25922 *	22	24	10	20	16	15
*P. aeruginosa ATCC 27853 *	23	20	13	20	18	17

Compound	Clotrimazole	**4d**	**4e**	**4f**	**4g**	**4h**

Test fungi						
*C. albicans ATCC 10231 *	20	21	16	18	17	17
*A. flavus ATCC 204304 *	22	20	17	17	18	15
*A. niger ATCC 16404 *	21	19	16	20	16	16
